# Advances in Coffee Drying: A Comprehensive Review of Traditional, Solar, Mechanical, Hybrid, and Emerging Methods

**DOI:** 10.3390/foods15101737

**Published:** 2026-05-14

**Authors:** Eduardo Duque-Dussán, Paula A. Figueroa-Varela, Valentina Cruz-Ospina, Jan Banout

**Affiliations:** 1 Engineering Discipline, National Coffee Research Center—Cenicafé, km 4, Vía Antigua Chinchiná—Manizales, Manizales 170008, Caldas, Colombia; eduardo.duque@cafedecolombia.com (E.D.-D.); valentina.cruz@cafedecolombia.com (V.C.-O.); 2 Plant Breeding Discipline, National Coffee Research Center—Cenicafé, km 4, Vía Antigua Chinchiná—Manizales, Manizales 170008, Caldas, Colombia; paula.figueroa@cafedecolombia.com; 3 Department of Food and BioResource Technology, Faculty of Tropical AgriSciences, Czech University of Life Sciences Prague, Kamycka 129, 16500 Prague–Suchdol, Czech Republic

**Keywords:** cup quality, energy efficiency, heat and mass transfer, moisture diffusion, postharvest processing, renewable energy, smallholder adoption

## Abstract

Drying is a critical stage in the postharvest chain, shaping product stability, quality, and economic value. Freshly harvested beans contain high moisture, and inadequate drying can lead to microbial growth, physical deterioration, and loss of key sensory attributes. In recent decades, diverse technologies have been developed to enhance drying efficiency while preserving flavor, improving consistency, and reducing environmental impacts. This review adopts a systematic and comparative approach, synthesizing peer-reviewed literature on conventional practices, advanced solar dryers, mechanical systems, hybrid configurations, and emerging techniques such as microwave, infrared, and desiccant-assisted drying. Emphasis is placed on heat and mass transfer mechanisms, the influence of environmental and operational parameters, and the role of varietal and processing differences. Comparative analyses reveal trade-offs between energy consumption, drying kinetics, and impacts on physical and chemical quality. Sustainability aspects are also examined, including energy use, carbon footprint, water consumption, and scalability for smallholders. Finally, key research gaps are identified, particularly in multiscale modeling, real-time monitoring, and integration with renewable energy and smart control systems. The review highlights pathways for achieving greater consistency, lower environmental burdens, and stronger value chains in producing regions worldwide.

## 1. Introduction

Coffee is one of the most important agricultural commodities worldwide, providing livelihoods for more than 25 million smallholder farmers across Africa, Asia, and Latin America and ranking among the most valuable agricultural products in international trade [1,2,3,4]. Once harvested, coffee cherries undergo a sequence of postharvest operations designed to transform the fresh fruit into stable, high-quality beans suitable for long-distance transport, storage, and roasting. These operations—sorting, pulping, fermentation, washing, drying, and storage—collectively determine the physical integrity, chemical composition, and sensory expression of the beans, which ultimately define their market value and consumer acceptance [5,6,7].

Within this chain, drying emerges as the most critical and often most challenging operation. Freshly harvested beans typically contain 50–60% moisture on a wet basis, depending on the processing method and local climatic conditions, and this must be reduced to safe storage levels of 10–12% (wb) [8,9,10]. Achieving this reduction is essential to inhibit enzymatic activity, microbial growth, and the onset of mycotoxin contamination, particularly ochratoxin A, which poses serious risks to food safety [11,12]. Inadequate or poorly managed drying not only compromises safety but also accelerates biochemical degradation, resulting in discoloration, uneven fermentation, and the loss of volatile compounds that contribute to flavor complexity and aroma [13,14,15]. Because of these combined effects, drying should be understood not merely as a preservation step but as a critical bottleneck that directly links farm-level practices with quality outcomes and compliance with global market standards [16,17].

Despite the wide range of drying technologies currently available, significant challenges remain in achieving consistent, efficient, and sustainable coffee drying across diverse production systems. Traditional methods, such as open sun drying and basic solar systems, are highly dependent on climatic conditions, leading to prolonged drying times, uneven moisture distribution, and increased risks of microbial contamination and quality deterioration [5]. Conversely, mechanical drying technologies provide greater control and faster processing but are often associated with high energy consumption, elevated operational costs, and reliance on fossil fuels, raising concerns about environmental impact and economic feasibility, particularly for smallholder farmers. In addition, increasing market demands for high-quality and specialty coffee, coupled with stricter sustainability standards and climate variability, have exposed the limitations of existing drying practices. These challenges highlight a critical gap between current drying technologies and the evolving requirements of the coffee sector, underscoring the need for more efficient, adaptable, and environmentally sustainable drying solutions.

**Figure 1:**
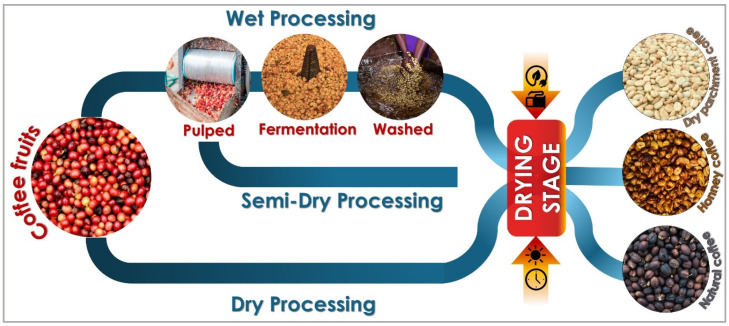
Schematic overview of post-harvest coffee processing pathways (wet, semi-dry, and dry methods), highlighting the critical role of the drying stage.

Coffee postharvest processing directly determines both economic value and sensory quality [2,18]. Three processing pathways dominate production: natural, honey, and washed. Each method differs in handling of the cherry and mucilage, yet all converge on drying as the decisive stage for quality, safety, and long-term stability (Figure 1) [19,20,21].

In the natural process, whole cherries are dried with pulp and mucilage intact [20,22]. This procedure can extend over three weeks under favorable climatic conditions and requires constant monitoring to avoid uneven fermentation, mold development, or sensory defects such as sour or over-fermented notes [13,23]. When managed effectively, the natural process produces coffees with distinctive attributes, including enhanced sweetness, heavier body, and increased complexity—characteristics highly valued in specialty markets [24,25].

The honey, or pulped natural, process is an intermediate approach. Here, the skin is removed, but varying amounts of mucilage are retained, producing categories such as yellow, red, or black honey, each associated with specific drying dynamics, stickiness, and microbial susceptibility [21,26]. Honey coffees dry faster than the natural ones but still require strict control of layer thickness, frequent turning, and adequate ventilation to prevent clumping and localized fermentation [7,27].

By contrast, the washed process removes most mucilage by fermentation and washing before drying [28,29]. This yields flavor profiles that are cleaner and more consistent, making washed coffees particularly desirable in international markets [30,31]. Although less prone to fermentation defects, washed beans are vulnerable to reabsorbing moisture in humid environments, which can compromise stability and increase brittleness during storage [5,32]. Despite these methodological differences, drying remains the universal bottleneck in postharvest processing, ultimately determining whether coffee achieves its full sensory and commercial potential [10,33,34].

Drying governs not only moisture reduction but also critical biochemical and physical transformations [35,36,37,38]. The rate and uniformity of water loss influence the stability of organic acids, sugars, proteins, and lipids that shape cup attributes such as acidity, sweetness, body, and mouthfeel [39,40]. Preservation of volatile precursors, including aldehydes, ketones, and phenolics, is equally sensitive to drying kinetics; uneven or rapid drying can degrade delicate aromatics, diminishing fruity or floral notes [41,42,43]. Structural integrity of the parchment and endosperm is also established during drying, with direct implications for roasting performance, storage stability, and color uniformity [44,45]. Poorly managed drying often results in hidden defects that later manifest as moldy, earthy, or phenolic off-flavors, reducing commercial grade, while controlled drying ensures consistency, preserves terroir, and safeguards quality [5,46].

Food safety considerations further underscore the importance of drying. Reducing water activity suppresses microbial growth and prevents contamination by *Aspergillus* and *Penicillium* species capable of producing ochratoxin A [11,47]. This mycotoxin is among the most serious safety concerns in coffee and is strictly regulated in major importing markets [48]. In tropical regions, where relative humidity frequently exceeds 70% during harvest, inadequate ventilation, rain interruptions, or delays in drying increase the risk of fungal colonization if moisture content remains above 12% (wb) [11,49,50]. Such conditions jeopardize both quality and compliance, with potential rejection in international trade. Controlled drying is therefore indispensable for ensuring consumer safety, maintaining market access, and protecting producer livelihoods [51,52].

Drying performance and its outcomes are further influenced by environmental, biological, and operational factors. Temperature, humidity, and airflow regulate the dynamics of moisture removal, while the thickness of coffee layers and frequency of turning determine uniformity [53,54]. Bean properties such as species, variety, and processing pathway introduce additional variability; for example, Arabica requires tighter control than Robusta, and natural, honey, or washed coffees each pose distinct risks of microbial growth or sensory defects [21,55]. Failures in management, including re-wetting or case hardening, compromise stability and cup quality, whereas well-controlled drying preserves organic acids, sugars, and volatile precursors, enabling superior sensory expression [5]. Because these dynamics extend into storage and roasting, the effectiveness of drying practices directly predicts not only food safety but also consistency, shelf life, and the commercial grade of coffee [16,56,57].

The limitations of conventional practices have stimulated the development of new technologies. Open-sun drying remains the most widespread due to its simplicity and low cost, but it requires extensive surfaces, is weather-dependent, and often produces inconsistent results [5,58]. Mechanical dryers allow for faster and more uniform drying under controlled airflow and temperature but are constrained by high operational costs, fossil fuel dependence, and environmental impacts that restrict their adoption by smallholder farmers [59,60].

To address these challenges, intermediate and innovative solutions have emerged. Solar dryers, using passive or active ventilation, reduce reliance on favorable weather while harnessing renewable energy [61,62]. Hybrid systems that integrate solar energy with mechanical or biomass heat sources improve resilience under variable climates [63,64]. More recently, advanced systems incorporating photovoltaic panels, automation, and sensor-based monitoring have been proposed to optimize energy efficiency, stabilize drying kinetics, and lower labor demands [65,66]. Collectively, these developments reflect the sector’s drive to reconcile quality and safety with sustainability and accessibility [67,68].

Despite the growing body of research on coffee drying, existing studies are often fragmented, focusing on specific technologies, isolated process variables, or limited quality attributes. Many investigations emphasize drying kinetics or equipment performance under controlled conditions, while fewer studies integrate these aspects with energy consumption, environmental impact, and real-world applicability across different production scales. In addition, the influence of climatic variability and its interaction with drying technologies remains insufficiently addressed, particularly in the context of smallholder systems where environmental and economic constraints are critical. As a result, there is a lack of comprehensive frameworks that simultaneously evaluate drying technologies in terms of technical performance, product quality, sustainability, and scalability. Addressing this gap is essential to support more informed technology selection and to align drying practices with the evolving demands of the coffee sector.

In summary, drying is the pivotal stage that links agricultural practices with consumer expectations [69]. It shapes sensory expression, guarantees food safety, and ensures compliance with international standards, while being increasingly challenged by climate variability, energy costs, and sustainability imperatives [70]. Drying is therefore not only a preservation step but also a decisive control point linking postharvest management with quality, safety, energy use, and producer income. In this context, the present review provides an integrated assessment of coffee drying technologies across traditional, solar, mechanical, hybrid, and emerging systems. Unlike previous reviews that address only selected technologies or broader food-drying principles, this review synthesizes drying physics, technological performance, impacts on sensory and physical quality, sustainability implications, and adoption constraints across different production scales. Particular attention is given to trade-offs among drying time, energy demand, climate resilience, product quality, and feasibility for smallholder and industrial contexts. By combining these dimensions, the review aims to support both future research and more informed decision-making in coffee-producing regions.

### Scope and Literature Selection

This article was prepared as a narrative review with a structured literature selection strategy. A targeted search was conducted in major scientific databases, including Scopus, Web of Science, and Google Scholar, covering publications from 2022 to 2026. The search combined keywords such as “coffee drying”, “postharvest coffee processing”, “solar drying”, “mechanical drying”, “drying kinetics”, “heat and mass transfer”, “coffee quality”, and “energy efficiency”, along with terms related to emerging technologies (e.g., “microwave drying”, “infrared drying”, “freeze-drying”, and “desiccant drying”).

The search considered peer-reviewed journal articles, book chapters, technical reports, and selected conference contributions relevant to coffee postharvest processing and drying engineering. Preference was given to experimental and review studies with clear methodological descriptions and quantitative results and to studies addressing drying kinetics, heat and mass transfer, energy performance, impacts on bean quality and safety, and applicability under different production scales and climatic conditions. Additional references from broader food-drying literature were included only when they contributed transferable engineering principles or methodological insights relevant to coffee drying. The objective was not to conduct a formal systematic review or meta-analysis; rather, the aim was to synthesize current knowledge, identify consistent trends, highlight technology-specific trade-offs, and outline key research gaps relevant to both scientific research and practical implementation.

## 2. Traditional Coffee Drying Methods

Traditional drying methods remain the dominant approach for moisture reduction in coffee across most producing regions, particularly among smallholder farmers in Latin America, Africa, and Asia [58,59,71]. These systems are characterized by their reliance on direct solar radiation and ambient airflow as the primary drivers of heat and mass transfer, which makes them inherently low-cost, low-technology, and accessible to resource-limited producers [72,73]. In addition to their affordability, these systems are frequently regarded as environmentally favorable because they operate without fossil fuel inputs and produce no direct greenhouse gas emissions [74].

From a physical perspective, drying under natural conditions is governed by the interaction between solar irradiance, ambient temperature, relative humidity, and wind speed, all of which are highly variable across time and location [49,75]. Such dependence introduces strong heterogeneity into drying kinetics, as beans may undergo rapid evaporation during midday but reabsorb moisture at night or during periods of high humidity. This variability extends the overall drying cycle, which can last from 7 to 20 days depending on climate and processing method [76,77]. Prolonged exposure also increases the risk of microbial proliferation, particularly fungal species such as *Aspergillus* sp. and *Penicillium* sp., capable of producing ochratoxin A under high humidity conditions [78,79].

Despite these limitations, traditional drying methods continue to be widely used due to their simplicity, scalability at the farm level, and cultural embeddedness in coffee-producing communities [62,80]. In many regions, patios, raised beds, and parabolic houses represent not only technical choices but also socio-economic adaptations to local constraints in infrastructure and capital investment. However, these methods are also closely associated with variability in quality outcomes: uneven drying, case hardening, or re-wetting events can lead to inconsistent cup profiles and reduced storage stability [5,63]. For these reasons, traditional methods occupy a paradoxical position in coffee postharvest systems.

### 2.1. Patio Drying

**Figure 2:**
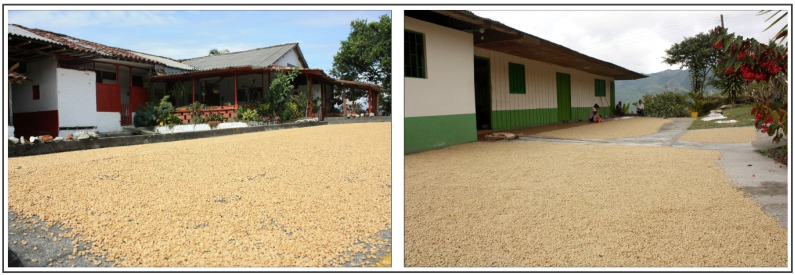
Traditional open sun drying of parchment coffee on concrete patios.

Patio drying is one of the oldest and most widely adopted postharvest methods for coffee processing, particularly in Latin America, Africa, and parts of Asia [58,81]. In this system, coffee is spread in thin layers (2–5 cm) over flat surfaces such as cement, brick, or asphalt and exposed directly to solar radiation and ambient airflow [82,83] (Figure 2). Drying depends entirely on natural heat input and convective mass transfer, and beans are turned periodically to improve uniformity and reduce localized fermentation [84,85]. Depending on climatic conditions, drying typically requires 7–20 days and may be interrupted by rainfall or high relative humidity, increasing the risk of re-wetting [49].

From a physical perspective, patios enable direct solar absorption at the bean surface and conductive heat transfer from the heated floor material, which can substantially exceed ambient temperature during peak radiation [72,86]. While this accelerates daytime evaporation, pronounced diurnal fluctuations may promote nocturnal moisture reabsorption, increasing heterogeneity in final moisture content and the risk of fungal contamination, including ochratoxin A–producing *Aspergillus* spp. [11,48]. Additionally, the open configuration exposes coffee to environmental contaminants unless strict hygiene practices are applied.

Patio drying offers low capital and maintenance costs, operational simplicity, and the capacity to process large volumes simultaneously, which explains its persistence among smallholder producers [80]. However, it requires extensive land area, is highly climate-dependent, and generally exhibits slower and more variable drying kinetics than raised beds or solar dryers [58]. This variability can increase the incidence of moisture heterogeneity and associated sensory defects [5]. Nevertheless, under stable dry climates and with appropriate management of layer thickness and turning frequency, patio drying can achieve acceptable moisture targets and satisfactory cup quality.

### 2.2. Raised Beds

**Figure 3:**
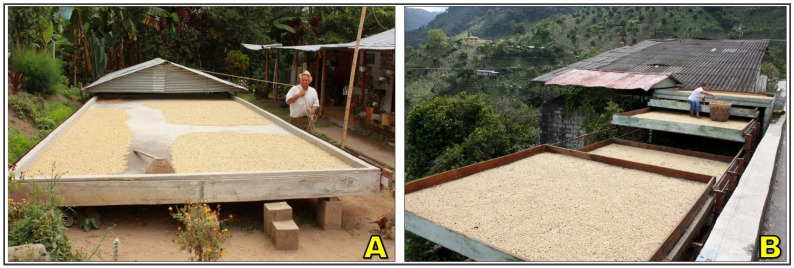
Typical small-scale movable drying structures: (**A**) Sliding-roof platform (casa-elda) and (**B**) multi-tier sliding tray system. Photographs taken by the authors; images are provided as representative examples of commonly used small-scale drying systems.

Raised beds, also referred to as African beds or drying tables, are elevated platforms designed to enhance airflow and reduce contamination risks during coffee drying [13] (Figure 3). They are typically built from wooden or metal frames and covered with a mesh or netting surface that supports the beans while allowing air to circulate both above and below [87]. Standard heights range from 0.8 to 1.2 m, with coffee spread in layers of 2–4 cm to facilitate uniform exposure to solar radiation and convective airflow [88]. As with patios, frequent turning, at least 2–3 times per day, is necessary to prevent clumping, reduce microbial growth, and minimize localized fermentation.

Raised-bed drying improves convective mass transfer by allowing airflow beneath the coffee beans, which accelerates evaporation, promotes more uniform moisture removal, and reduces the formation of anaerobic conditions associated with undesirable fermentation [73]. By elevating the beans above ground level, this system also minimizes contamination from soil, dust, animals, and water, making it more hygienic than patio drying and reducing the risk of fungal growth and ochratoxin formation [89]. Additionally, enhanced ventilation enables higher drying efficiency per unit area and contributes to improved preservation of sensory quality, including greater flavor clarity, fewer off-notes, and higher cupping scores in specialty markets [71]. However, raised beds involve higher construction and maintenance costs, increased labor demands, and limited scalability in some contexts, and they remain vulnerable to rainfall and nocturnal moisture reabsorption if not adequately protected [88].

### 2.3. Parabolic Houses

**Figure 4:**
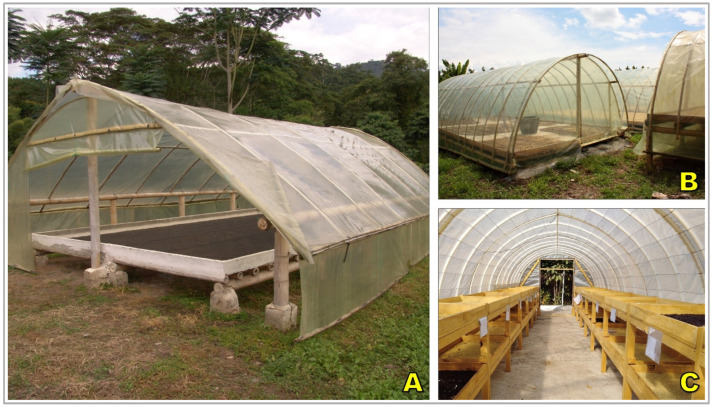
Different configurations of parabolic greenhouse-type solar dryers: (**A**) Low-profile parabolic dryer with raised concrete bed, (**B**) high-arched passive solar tunnels, and (**C**) internal view of a multi-tier greenhouse dryer illustrating vertical space utilization for batch drying.

Parabolic houses, also referred to as solar tents or greenhouse dryers, represent a semi-controlled environment for coffee drying designed to mitigate the vulnerabilities of open-air systems [86,90]. These structures typically consist of arched metallic or wooden frames covered with transparent polyethylene or polycarbonate sheets (Figure 4). The cover creates a greenhouse effect, allowing solar radiation to pass through while trapping longwave infrared radiation, which increases internal air temperature by approximately 5–15 °C above ambient conditions [91,92]. Coffee is spread either directly on the ground surface, usually lined with plastic or cement (Figure 4A,B), or on raised mesh tables inside the tunnel (Figure 4C). To avoid excessive heat and moisture accumulation, ventilation openings are strategically positioned along the sides, and in more advanced designs, fans are incorporated to regulate airflow and temperature [83,90,93].

From a functional perspective, parabolic houses provide protection from rainfall and high ambient humidity, thereby reducing interruptions in the drying process. They also accelerate drying by maintaining higher internal temperatures and stabilizing airflow conditions, which reduces exposure to microbial proliferation and fermentation defects [94,95]. The controlled environment improves consistency in drying kinetics, often shortening total drying time compared to patios or raised beds [13].

Despite their recognized advantages, parabolic houses present notable technical and economic limitations, including higher initial construction costs than patios or raised beds; recurring maintenance due to UV-induced degradation of plastic or polycarbonate covers; and potential drying problems associated with poor ventilation design, such as overheating, case hardening, internal condensation, and uneven airflow distribution [62,96]. These constraints can restrict adoption by resource-limited producers and often require cooperative schemes or external support to be economically viable. Nevertheless, field evaluations indicate that parabolic houses can reduce drying times by approximately 20–40% relative to open patios, while achieving more uniform final moisture content, improved storage stability, and a lower incidence of fungal-related defects, including reduced ochratoxin A contamination [97]. Sensory assessments further suggest enhanced flavor consistency, fewer off-notes, and higher overall cup scores compared with fully exposed drying systems, positioning parabolic houses as an effective intermediate option between traditional sun drying and capital-intensive mechanical drying technologies [94].

## 3. Solar Dryers

While traditional drying methods such as patios, raised beds, and parabolic houses continue to dominate coffee postharvest systems due to their low cost and reliance on free solar energy, their performance is highly constrained by climatic variability, long drying times, and exposure to contamination risks [5,98]. Solar energy, however, remains an abundant, renewable, and cost-free resource in tropical coffee-growing regions, and efforts to improve its efficiency and reliability have given rise to the development of solar dryers [62,99]. These systems aim to harness solar radiation while creating a more controlled microclimate that reduces dependence on weather conditions and improves drying consistency. By integrating structural enclosures, selective covers, and sometimes mechanical assistance, solar dryers combine the low-cost advantage of traditional methods with enhanced control of heat and mass transfer processes.

Solar dryers can be broadly classified into passive systems, which rely on natural convection to move air through the drying chamber, and active systems, which incorporate forced ventilation to increase airflow and heat distribution [100,101]. Within these categories, diverse designs have been developed, including tunnel dryers, greenhouse-type dryers, and polycarbonate-covered enclosures, each with unique construction requirements, drying efficiencies, and implications for coffee quality [89].

This chapter reviews the main types of solar dryers, beginning with passive designs, then examining active systems with forced-air ventilation, and finally summarizing experimental evaluations and comparative performance reported in the literature.

### 3.1. Passive Solar Dryers

**Figure 5:**
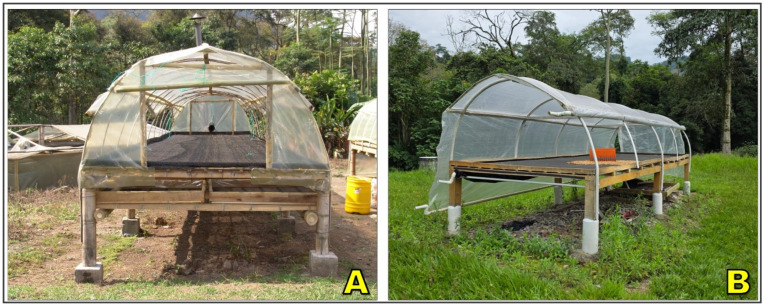
Comparative structural configurations of passive solar tunnel dryers for parchment coffee: (**A**) A sustainable framework constructed from *Guadua angustifolia Kunth* (structural bamboo) clad with UV-stabilized low-density polyethylene (LDPE) greenhouse-grade film; (**B**) a lightweight polyvinyl chloride (PVC) vaulted frame featuring woven poly-laminate (scrim-reinforced) fabric.

Passive solar dryers operate without mechanical assistance, using the heat transfer that occurs by natural convection, where the air movement is caused by differences in density due to the thermal changes inside the drying chamber and the environment [102]. Typically designed as tunnel or greenhouse-type structures covered with polyethylene or polycarbonate sheets, they allow solar radiation to enter and raise the internal air and bean temperature (Figure 5). The resulting density gradients sustain continuous airflow without fans, creating a protected microenvironment that shields coffee from rain, dust, and pests while maintaining temperatures commonly 5–20 °C above ambient [103].

This thermal gain accelerates moisture removal and reduces total drying time by approximately 20–40% compared with open patios, shortening cycles from 15 to 20 days to roughly 7–12 days under similar climatic conditions [76,103]. Although passive dryers provide protection from rainfall and external contamination, nocturnal re-wetting and internal condensation may still occur under inadequate ventilation or high nighttime humidity, potentially affecting moisture uniformity and microbial stability [17,71,104]. Performance, however, depends strongly on structural design, vent configuration, and local wind conditions, as airflow is governed exclusively by natural convection [49].

Material selection further influences durability and thermal efficiency. Polyethylene covers are inexpensive but degrade relatively quickly under UV exposure, often requiring replacement within a few years, whereas polycarbonate sheets offer greater longevity, improved UV resistance, and better heat retention [76,99,105]. Despite sensitivity to design and ventilation, passive solar dryers remain an effective intermediate technology between traditional sun drying and mechanically assisted systems, combining low operational costs with enhanced environmental control and improved drying consistency.

### 3.2. Active Solar Dryers

Active solar dryers are designed to overcome the airflow limitations of passive systems by incorporating mechanical assistance, typically through fans or blowers powered by electricity, photovoltaic panels, or small engines [89,106]. In these systems, solar radiation is still the primary source of heat, but forced-air ventilation improves the distribution of thermal energy and enhances mass transfer between the bean surface and the drying air [107].

**Figure 6:**
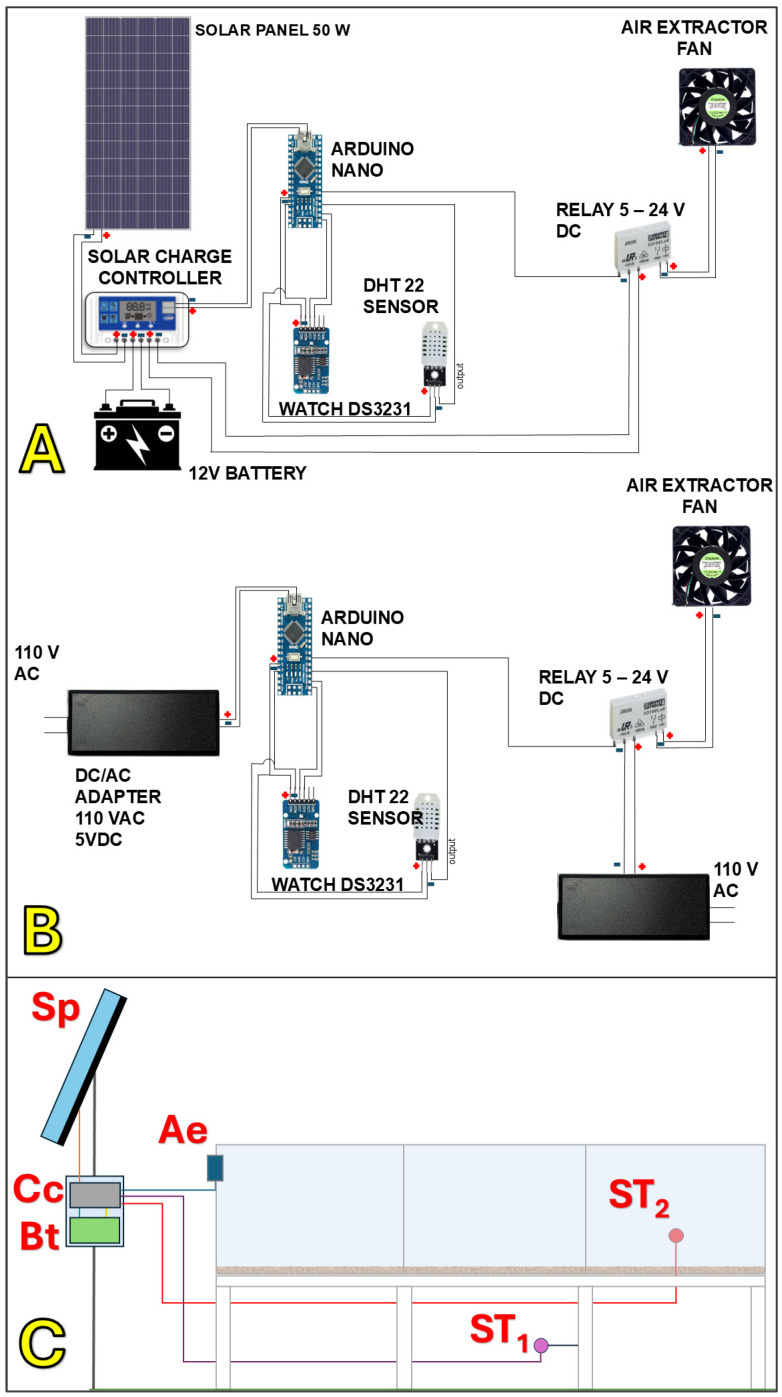
Electronic control architectures for autonomous moisture removal in solar drying: (**A**) Off-grid configuration powered by a 50 W photovoltaic panel with battery storage; (**B**) grid-connected configuration using an AC/DC adapter; and (**C**) schematic representation of the automated greenhouse dryer. The system components include: Sp (Photovoltaic Solar Panel), Cc (Charge Controller Unit), Bt (Battery Bank), Ae (Air Extractor/Fan), and ST_1_–ST_2_ (Hygrothermal Sensors for internal and external temperature/humidity monitoring). The integration of an Arduino Nano microcontroller (Arduino, Turin, Italy) and DHT22 sensors (Aosong Electronics Co., Ltd., Guangzhou, China) allows for intelligent actuation of forced convection based on real-time environmental data [62].

Active solar dryers typically incorporate solar collectors such as flat-plate or integrated roof panels that capture and concentrate solar energy to preheat incoming air, which is then circulated across the coffee bed by fans or blowers to ensure more uniform temperature and humidity conditions [100,107,108] (Figure 6). Compared with passive systems, these designs achieve higher drying rates, shorter processing times, and improved control of final moisture content, with reported temperature increases of 10–25 °C above ambient and drying-time reductions of 30–50% relative to patios or passive tunnels, depending on system configuration and weather conditions [62,98,109]. A key advantage is their reduced dependence on external climatic variability, as forced airflow maintains consistent drying conditions, limits nocturnal moisture reabsorption, and lowers the risk of uneven drying, fungal growth, and microbial contamination [110]. Nevertheless, active dryers entail higher construction and operating costs due to the inclusion of fans, collectors, and energy requirements, and although photovoltaic systems can offset energy use, they further increase capital and maintenance costs. Additionally, inadequate system design may result in overheating, case hardening, excessive energy consumption, or deterioration of sensory quality [62,111].

### 3.3. Experimental Evaluations and Comparative Performance

**Figure 7:**
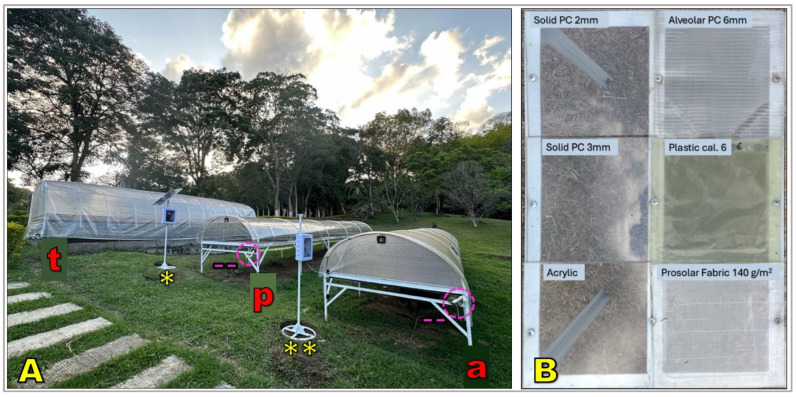
Comparative evaluation of greenhouse-type solar dryers and cladding substrates. (**A**) Experimental field setup showcasing three structural configurations: t (traditional system with flexible LDPE film), p (solid polycarbonate cover), and a (alveolar polycarbonate cover). (**B**) Detailed array of cladding specimens under analysis, including solid Polycarbonate Cover (PC) (2 mm, 3 mm), alveolar PC (6 mm), acrylic, greenhouse-grade plastic (cal. 6), and reinforced poly-laminate fabric (140 g/m^2^). The integrated photovoltaic (PV) and sensing units, marked with * and **, facilitate real-time monitoring of radiative transmittance and internal hygrothermal dynamics [62]. Dashed purple circles—display nighttime operation rollable plastic cover to avoid product moisture reabsorption.

The performance of solar dryers has been extensively evaluated under experimental and field conditions (Figure 7), consistently demonstrating higher efficiency and improved quality preservation compared to traditional open-air drying. Both passive and active systems reduce drying times by 20–50% relative to patios and raised beds, depending on design, climate, and initial moisture content [58,103]. Tunnel-type dryers covered with polyethylene or polycarbonate typically shorten drying cycles from 15 to 20 days to 7–12 days, while active systems with forced ventilation and solar collectors may reduce drying to 4–7 days [62,63,89].

Beyond shorter drying times, solar dryers improve uniformity of final moisture content, enhancing storage stability and reducing batch heterogeneity [17]. Greater consistency lowers the risk of defects such as moldy, earthy, or phenolic notes associated with uneven drying and microbial contamination [112,113]. Several studies report higher cupping scores, particularly in cleanliness, acidity, and balance, for coffee dried in solar dryers compared to patios [42,103,114]. Reduced exposure to dust, soil, and rain also improves food safety and decreases the likelihood of ochratoxin A contamination [11,78,115].

Material selection and system configuration further influence performance. Polycarbonate covers provide greater durability, UV resistance, and thermal retention than polyethylene, promoting more stable drying conditions and lower maintenance needs [116,117,118]. Systems with adjustable vents or hybridized with small fans allow better airflow and temperature control, improving drying uniformity [89,119,120]. Nevertheless, passive dryers may still present uneven airflow and localized moisture gradients [121,122], whereas active systems require higher capital and operating costs, which can limit adoption among smallholders (Table 1) unless supported through subsidies or cooperative models [123,124].

The comparison shows a progressive improvement in performance as systems evolve from traditional open-air drying to more controlled solar technologies. Patios and raised beds remain prevalent due to low cost and simplicity but are strongly constrained by weather variability, longer drying periods, and higher contamination risk. Passive solar dryers mitigate part of this variability by creating a protected microclimate, reducing drying time and improving quality consistency. Active systems provide the highest level of control, achieving shorter drying cycles, greater airflow and temperature regulation, and improved safety outcomes, although their higher capital and operating costs may limit adoption among smallholders. Drying conditions influence not only moisture removal kinetics but also the physicochemical stability of the coffee matrix. In particular, temperature, drying rate, and exposure time affect enzymatic activity, degradation of thermolabile compounds, and the development of precursor compounds that ultimately shape the sensory profile of the beverage.

## 4. Mechanical Dryers

Mechanical drying technologies were introduced to address the limitations of traditional and solar-based systems, particularly their dependency on weather conditions, long processing times, and vulnerability to microbial contamination [5,125]. These systems rely on controlled heating and forced airflow to accelerate moisture removal, allowing drying to be completed in 24–72 h depending on batch size, bean moisture, and dryer design [126,127]. By decoupling the process from environmental variability, mechanical dryers provide greater consistency, scalability, and year-round availability for coffee processing.

The development of mechanical dryers has been closely linked to the need for more reliable postharvest systems in regions where high humidity and frequent rainfall disrupt sun-based methods [128,129]. In addition, the increasing demand for uniform quality and food safety in international markets has made mechanical drying a central component of modern coffee supply chains. However, these benefits come at the cost of significant energy inputs, which directly affect operational costs and environmental footprint [64,65,130].

Mechanical dryers are broadly categorized into static bed dryers, cross-flow dryers, rotary dryers, and drum dryers, each with specific design principles and suitability for different production scales [131,132]. Beyond their typological differences, mechanical systems raise questions of energy balance, fuel efficiency, and carbon footprint, which are critical in the context of sustainable coffee production. Equally important are their impacts on sensory quality, since high drying temperatures or uneven heat distribution can alter bean chemistry and compromise cup attributes [44,133,134].

This chapter reviews the main typologies of mechanical dryers, analyzes their energy requirements and operational costs, evaluates their effects on quality, and considers their applications across both smallholder and industrial coffee production systems.

### 4.1. Static Bed Dryers

**Figure 8:**
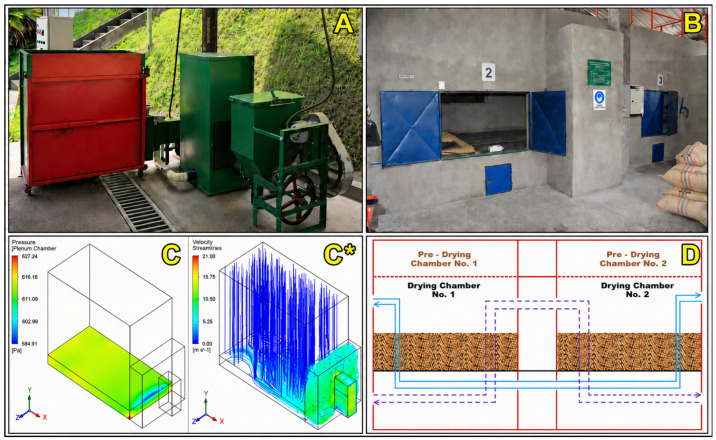
Engineering, architecture, and computational fluid dynamics (CFD) analysis of mechanical coffee dryers. (**A**) Experimental prototype of a mechanical drying unit; (**B**) industrial-scale Cenicafé-type mechanical dryer (Cenicafé, Manizales, Colombia) for parchment coffee; (**C**) CFD-derived profiles of static pressure distribution and (C*) velocity streamlines for the experimental unit A; (**D**) operational schematic illustrating the airflow distribution circuit within the Cenicafé-type dryer, highlighting the serial pre-drying and drying stages for enhanced thermal efficiency.

Static bed dryers are among the most widely used mechanical drying systems for coffee, particularly in Latin America, where they were introduced to provide an alternative to patio drying during periods of high rainfall or humidity [73]. In these systems, parchment or cherry coffee is loaded in a fixed chamber or plenum, typically in layers of 20–40 cm thickness, through which heated air is forced vertically from bottom to top (up-flow) or top to bottom (down-flow) by a fan [126,127]. The drying chamber is usually constructed of perforated metal sheets or mesh floors to allow uniform air distribution across the coffee mass (Figure 8).

These dryers heat the drying air using diverse energy sources, including biomass furnaces, often fueled by coffee husk or wood, as well as gas or electricity, with operating temperatures typically maintained between 40 and 60 °C to balance drying efficiency and preservation of bean chemistry and sensory quality [16,125]. Drying is driven by forced convection, with heat transferred mainly through convective airflow and conduction between beans, while moisture removal occurs via internal diffusion and surface evaporation [135]. Because the coffee bed remains stationary, moisture gradients commonly develop along the airflow path, often requiring stirring or partial unloading to improve drying uniformity [136]. These systems offer practical advantages such as relatively simple construction and operation, batch processing of moderate to large volumes, and independence from climatic conditions, particularly when integrated with on-farm biomass resources that enhance energy self-sufficiency and reduce operating costs [64,137]. Nevertheless, challenges persist, including non-uniform drying in thick beds, risks of case hardening or quality deterioration under prolonged or high-temperature conditions, and moderate energy efficiency due to high pressure drops and fan energy demands [127,136]. Despite these limitations, static bed dryers remain widely used in cooperatives and medium-sized farms handling approximately 250–2000 kg of parchment coffee per batch, owing to their robustness, simplicity, and compatibility with locally available biomass fuels [126]. 

### 4.2. Cross-Flow Dryers

**Figure 9:**
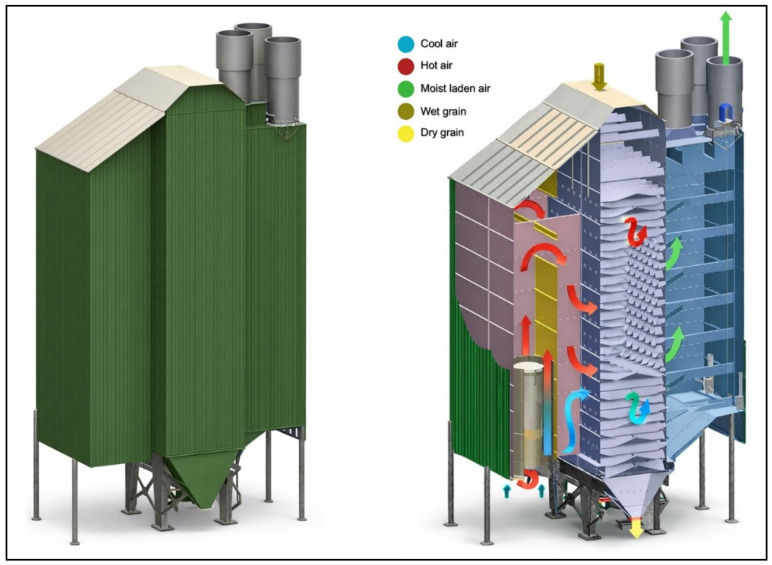
Structural and functional representation of a high-capacity cross-flow drying tower. (**Left**) External isometric view of the modular enclosure; (**Right**) Cutaway view revealing the internal heat exchange mechanism, where forced convective airflow crosses the vertical grain path to optimize the drying rate and thermal energy utilization in large-scale post-harvest operations [126].

Cross-flow dryers represent another important class of mechanical systems applied to coffee drying, particularly in regions where higher throughput and continuous operation are required [138,139]. Unlike static bed dryers, where air moves vertically through a fixed layer, cross-flow dryers are designed so that heated air passes horizontally across a moving or stationary bed of coffee, typically contained within vertical columns or cascading along perforated walls [140,141] (Figure 9).

In a common configuration, parchment coffee is loaded into tall drying columns, often 1 to 2 m in height, through which hot air is blown from one side to the other. As beans gradually descend by gravity from the top to the discharge outlet at the bottom, they are continuously exposed to lateral airflow, creating a cross-current interaction between the drying medium and the product [126,142]. This geometry enables larger capacities than static dryers, often handling several tons per cycle, and is suitable for semi-continuous or continuous operation in cooperative and industrial facilities.

Cross-flow dryers promote intense convective heat and mass transfer by directing lateral airflow across the coffee column, resulting in faster drying than static systems, with typical operating temperatures of 45–65 °C and drying times of approximately 24–48 h, depending on initial moisture and airflow conditions [140,143]. Their main advantages include high throughput, shorter drying cycles, high efficiency, suitability for continuous operation, reduced labor demands, and flexibility in the use of different heat sources such as biomass, oil, or liquefied petroleum gas (LPG) [144,145]. However, moisture non-uniformity remains a key limitation, as beans near the air inlet tend to dry more rapidly than those near the outlet, and high airflow resistance through dense beds increases fan energy consumption and operational costs [141,146]. If airflow and temperature are not properly controlled, these systems also present a higher risk of overheating and associated deterioration of bean chemistry and sensory quality. Consequently, cross-flow dryers are most commonly adopted in large cooperative and industrial processing facilities, where high capacity and reduced drying times justify their greater technical complexity and energy requirements [147].

### 4.3. Rotary Dryers

**Figure 10:**
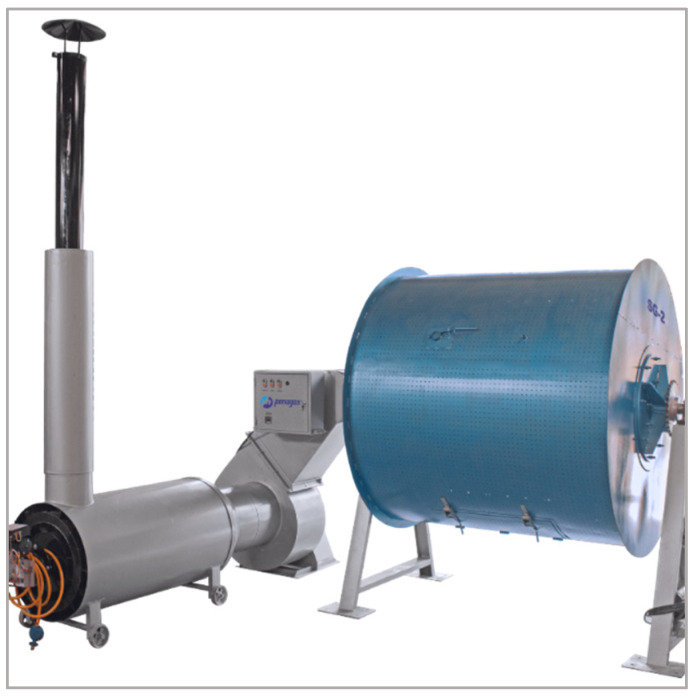
Industrial rotary drum dryer. Thermal energy generation unit featuring a chimney for combustion gas evacuation and a centrifugal fan for forced convection; a perforated rotary drum designed for batch or continuous operation. Penagos Hermanos. Coffee Processing Plants. Available online: https://penagos.com/centrales-de-procesamiento-de-cafe/ (accessed on 27 April 2026).

Rotary dryers are widely employed in large-scale coffee processing facilities where high throughput and continuous mixing of beans are required [147,148]. These systems consist of a rotating cylindrical drum, typically mounted horizontally or at a slight incline, that continuously agitates the coffee as it moves through the dryer [149,150]. Hot air is introduced either co-currently (flowing in the same direction as the beans) or counter-currently (opposite direction), depending on the design, to provide the thermal energy necessary for moisture removal, as seen in Figure 10 [10,38,151].

**Figure 11:**
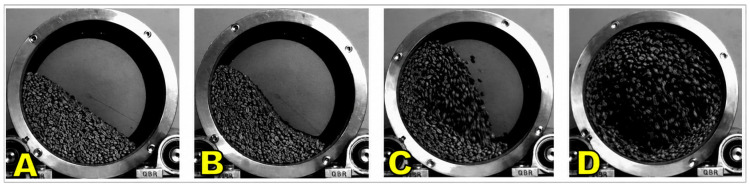
Visual characterization of bed motion in rotary coffee dryers. (**A**) Rolling, (**B**) Cascading, (**C**) Cataracting, (**D**) Centrifuging. [152].

The constant tumbling of the beans ensures uniform exposure to heated air, reduces the formation of localized moisture pockets, and minimizes the risk of case hardening compared to static or cross-flow systems [38,148]. As displayed in Figure 11, mechanical agitation also prevents clumping, which is particularly advantageous when drying natural or honey-processed coffees that contain residual mucilage or pulp, where stickiness can otherwise hinder airflow [152,153].

Rotary dryers operate under conditions that vary with processing stage and product type, typically using temperatures of 40–60 °C for parchment coffee and slightly lower values for natural cherries to prevent thermal damage, with residence times ranging from minutes to several hours depending on drum design and initial moisture content [38,149]. Their main advantages include the capacity to process large volumes, improved drying uniformity through continuous agitation, reduced labor requirements, and compatibility with continuous industrial-scale processing lines [147,154]. However, these systems are energy-intensive due to both air heating and drum rotation, require substantial capital investment and ongoing mechanical maintenance, and pose risks to quality preservation if temperatures and airflow are not carefully controlled, as excessive agitation and heat may accelerate volatile compound loss [36].

### 4.4. Drum Dryers

**Figure 12:**
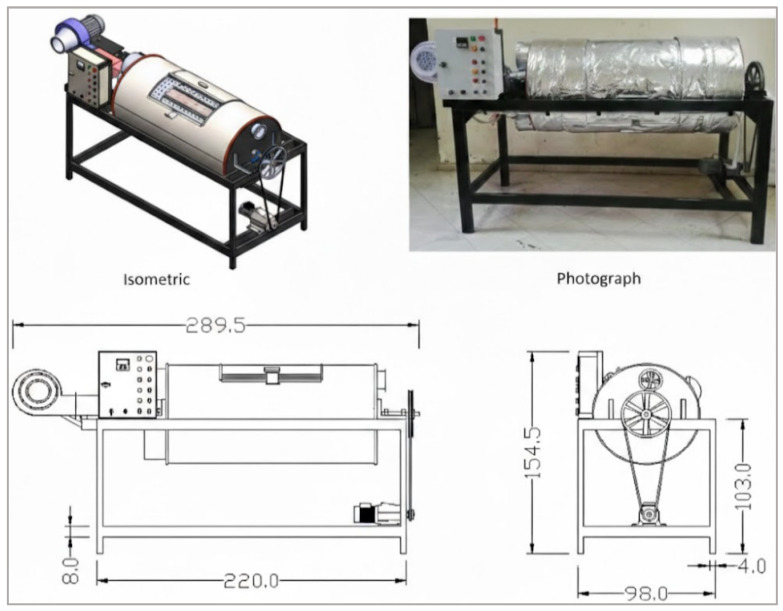
Engineering design of a pilot-scale rotary dryer: (Isometric/Orthographic) CAD projections detailing system dimensions and the physical prototype featuring a thermal insulation jacket and electronic control unit [151].

Drum dryers are a category of mechanical systems commonly used for coffee at small- to medium-scale operations, particularly in Latin America [155]. While they share similarities with rotary dryers in that beans are placed in a cylindrical chamber, drum dryers typically operate in batch mode and are designed to process smaller volumes (Figure 12), ranging from a few dozen kilograms up to one or two tons per cycle [144,156]. The drum, usually constructed of perforated metal, rotates slowly around its horizontal axis, ensuring that coffee is gently agitated during drying [157].

Heated air is introduced into the drum either directly, by channeling combustion gases through heat exchangers, or indirectly, by means of a furnace that supplies clean hot air via ducts [155,158]. Depending on the design, airflow may move co-currently with bean rotation or counter-currently, with typical operating temperatures between 40 and 55 °C to protect bean quality [132,159]. Residence times generally range from 12 to 36 h, depending on initial moisture content, drum capacity, and airflow rates [153,160].

From a process engineering perspective, drum dryers offer the advantage of mechanical mixing, which enhances drying uniformity compared to static bed dryers. Continuous agitation reduces the risk of localized over-drying or under-drying while also limiting microbial activity by preventing clustering of wet beans [152,153]. This makes them particularly effective for natural and honey-processed coffees, where residual mucilage and pulp increase stickiness and complicate drying in non-agitated systems [26].

Drum dryers offer a practical balance between capacity, robustness, and process control, providing more consistent drying than sun-based methods while remaining less complex and energy-intensive than large industrial rotary dryers [5]. Their compatibility with biomass fuels such as coffee husk or wood enhances sustainability and reduces operating costs, although energy consumption remains higher than in solar or hybrid systems and poor temperature control can affect sensory quality [149,161]. Because they operate in batch mode and require regular maintenance of moving parts, their throughput is lower than that of continuous systems. As a result, drum dryers are widely used in cooperatives and medium-sized farms as a reliable intermediate technology between traditional and fully industrial drying systems [155].

### 4.5. Energy Balance and Operational Costs

The efficiency and economic viability of mechanical coffee dryers are largely governed by their energy balance and associated operating costs. Drying is an energy-intensive process, typically requiring approximately 4000–5500 kJ per kilogram of water removed. These values represent general ranges derived from the literature and are intended to provide a broad overview of mechanical drying performance rather than figures associated with specific devices. Overall energy efficiency varies widely among dryer types, ranging from about 20–40% in conventional static and cross-flow systems to 40–60% in more optimized rotary and drum configurations. Such variability is largely influenced by system design and operating conditions, with energy losses commonly arising from inadequate insulation, excess airflow, and incomplete fuel combustion [60,136,149]. Factors such as combustion efficiency, thermal insulation, and process control therefore play a critical role in determining the overall energy performance of drying operations.

Fuel choice strongly influences both cost and sustainability: biomass residues such as coffee husk or parchment are widely used to reduce expenses and support circular energy use, although their variable physical properties can complicate combustion, while fossil fuels offer stable control at the expense of higher costs and carbon emissions [64,162,163].

In addition to thermal inputs, electrical energy for fans, blowers, and rotating components represents a significant share of total consumption, particularly in rotary and drum dryers, whereas static systems rely more heavily on fan power to overcome pressure drops in thick beds [60,63]. Consequently, operating costs reflect the combined effects of fuel use, electricity demand, and labor requirements, especially in batch-based systems, and may be two to five times higher than those of traditional sun-drying despite much shorter processing times [62,134,164]. To address these challenges, recent research has focused on energy optimization strategies such as improved furnace efficiency, heat recovery, variable-speed airflow control, and integration with solar pre-heating, aiming to lower costs and environmental impact while preserving drying performance and coffee quality [64,89,136,165].

### 4.6. Impact on Coffee Quality

The impact of mechanical drying on coffee quality is a subject of ongoing research, since drying parameters directly affect the chemical composition, physical integrity, and sensory attributes of the beans. While mechanical systems provide faster and more reliable moisture reduction compared to traditional or solar methods, their high energy inputs and intensive heat transfer can also alter the biochemical precursors that define cup quality [42,52,71].

From a chemical perspective, drying temperature and air velocity strongly influence the stability of organic acids, sugars, proteins, and lipids, which serve as precursors of flavor and aroma during roasting [166,167]. Excessive temperatures or prolonged exposure to high airflow rates can accelerate degradation of chlorogenic acids and volatile precursors, leading to reduced acidity, bitterness imbalances, and loss of aromatic complexity [125,168]. To prevent such damage, the maximum allowable drying temperature for coffee is generally considered to be 50 °C, as higher values increase the risk of chemical degradation and sensory deterioration [169,170]. Controlled drying within the range of 40–50 °C has been shown to best preserve desirable attributes while still achieving microbiological safety [171,172].

Physically, the rapid and uneven removal of moisture in mechanical systems may cause casehardening, a condition where the outer layers of the bean dry faster than the interior, creating a hard shell that traps residual moisture [44]. This phenomenon can result in internal cracking during storage or roasting, leading to heterogeneity in roast development and reduced cup uniformity. High airflow rates, while necessary for efficient drying, may also increase the risk of mechanical stress and breakage, particularly in thin-walled Arabica beans [125,173].

In terms of microbial safety, mechanical dryers have the advantage of reducing drying times to less than 72 h, which limits the window of fungal growth and ochratoxin A contamination [79,174]. However, poor control of air quality, recirculation of smoke from biomass furnaces, or uneven airflow distribution can compromise product safety and introduce undesirable smoky or phenolic notes [175].

Sensory studies have shown that coffees dried in well-managed mechanical systems can achieve comparable or even superior cupping scores to those dried traditionally, particularly in terms of cleanliness, uniformity, and shelf stability [5]. Nonetheless, reports also indicate that over-dried or heat-damaged coffees often exhibit lower complexity and muted terroir expression compared to those dried more slowly under controlled solar systems [125,173].

Ultimately, the quality outcomes of mechanical drying depend less on the technology itself than on the management of operational parameters, specifically temperature, airflow, loading density, and duration [16,126]. When optimized within the recommended maximum of 50 °C, mechanical dryers can preserve the intrinsic attributes of high-quality coffees while providing consistency and safety on a scale.

### 4.7. Applications in Small- and Large-Scale Systems

The adoption of mechanical coffee dryers varies markedly with farm scale, infrastructure, and market orientation, ranging from smallholder cooperatives to large industrial processing facilities. At the smallholder level, particularly in regions with high rainfall or prolonged harvest seasons, static bed and drum dryers are favored for their relative affordability, use of locally available materials, and compatibility with on-farm biomass fuels, typically handling 250–2000 kg of parchment coffee per batch and ensuring reliable attainment of safe moisture levels under adverse weather conditions [127,171]. In contrast, large-scale industrial mills rely predominantly on cross-flow and rotary dryers due to their high throughput, continuous operation, and integration with automated processing lines, especially in major producing countries where rapid, year-round processing is essential [10,38,176]. Despite their benefits in consistency and efficiency, adoption barriers persist; smallholders face high capital and fuel costs, often mitigated through cooperative ownership, while industrial facilities contend with substantial energy demands that drive interest in hybrid systems incorporating solar pre-heating or heat recovery. Overall, mechanical drying reflects a trade-off between accessibility and performance, with future developments likely focused on improving energy efficiency and cost-effectiveness while preserving coffee quality across different production scales [89,132].

## 5. Hybrid Drying Systems

**Figure 13:**
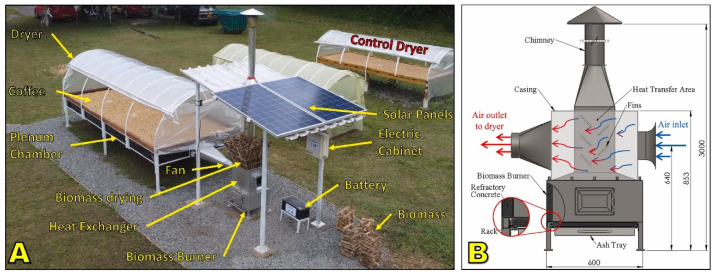
Hybrid Coffee Solar–Biomass Drying System: (**A**) Test assembly, which implements a tunnel-type solar drying chamber, a mechanical air heating system that uses coffee pruning waste (zoca) as fuel, integrated with a forced convection system powered by photovoltaics; (**B**) diagram of the biomass burner and heat exchanger assembly designed to maintain the drying temperature during periods of low or no solar radiation [63].

Hybrid drying systems have emerged as an intermediate solution between traditional/solar-based methods and fully mechanized dryers. While solar dryers offer low-cost operation and environmental sustainability, their performance is constrained by climatic variability and relatively long drying cycles. Conversely, mechanical dryers provide faster and more reliable moisture reduction but demand higher capital investment and significant energy inputs, often derived from fossil fuels [165,177,178]. To overcome these limitations, hybrid configurations (Figure 13) combine the advantages of solar energy with supplemental heat or airflow from mechanical or biomass-based systems [63,179].

The core rationale for hybrid dryers is to exploit the free and renewable nature of solar energy during periods of sufficient irradiance while providing backup heat or forced airflow when solar conditions are inadequate [110,180]. In this way, hybrid systems ensure both reliability and energy efficiency while minimizing operational costs and carbon footprint. Depending on the design, supplementary energy can come from electricity, biomass combustion, or integration with photovoltaic systems. Several hybrid typologies have been developed and tested:

- Solar–mechanical systems, which combine greenhouse-type dryers with fans or blowers powered by grid electricity or photovoltaic panels;
- Solar–biomass systems, where solar heating is supported by furnaces fueled with coffee husk, wood, or other residues;
- Solar–electric systems, which integrate solar collectors or panels with electric resistance heaters for temperature stabilization [108,180,181].

These systems have been reported to reduce drying time by 30–60% compared to purely passive solar dryers while improving uniformity of moisture removal and reducing microbial risks [63,100]. At the same time, they require less fuel and electricity than fully mechanical dryers, lowering operating costs and environmental impact.

This chapter reviews the main types of hybrid dryers, discusses their design principles and operational considerations, summarizes findings from experimental and field applications, and evaluates their scalability and economic feasibility in smallholder and industrial contexts.

### 5.1. Combinations of Hybrid Dryers

Hybrid dryers integrate solar energy with supplemental heat or airflow sources to enhance drying reliability and efficiency. Three major configurations are prominent in coffee processing: solar–mechanical, solar–biomass, and solar–electric systems [182,183].

#### 5.1.1. Solar–Mechanical Systems

**Figure 14:**
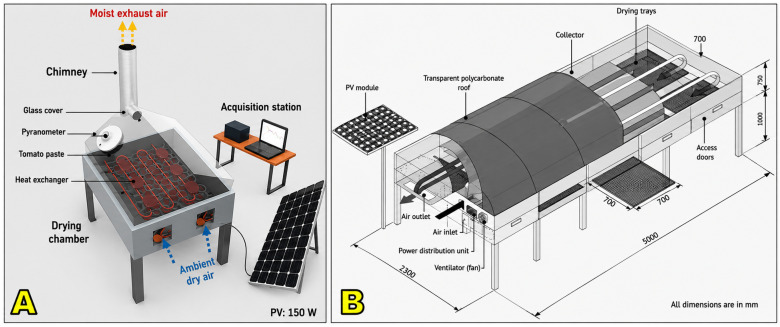
Solar–mechanical hybrid dryer designs. (**A**) Solar dryer with integrated heat exchanger in the form of electric resistors that heat the drying chamber [185]. (**B**) Solar dryer with integrated collector that heats the inlet air under the drying bed [186]. Both are powered by photovoltaics to move the fans.

Solar–mechanical hybrids combine the greenhouse effect of passive solar dryers with mechanical assistance in the form of fans or blowers, powered either by grid electricity or photovoltaic panels [61,110] (Figure 14). These systems maintain the protective structure of solar dryers while improving airflow distribution and convective heat transfer. By stabilizing air velocity and temperature inside the chamber, solar–mechanical dryers achieve faster and more uniform drying compared to passive systems. Typical drying cycles are reduced by 30–40%, while final moisture distribution is more consistent, reducing the risk of microbial contamination and postharvest defects [100,184].

However, the inclusion of fans introduces additional costs and energy requirements, which may be challenging for smallholder adoption unless supported by cooperatives or development programs [187,188]. Nevertheless, solar–mechanical systems are often regarded as an appropriate intermediate technology, balancing affordability with performance.

#### 5.1.2. Solar–Biomass Systems

**Figure 15:**
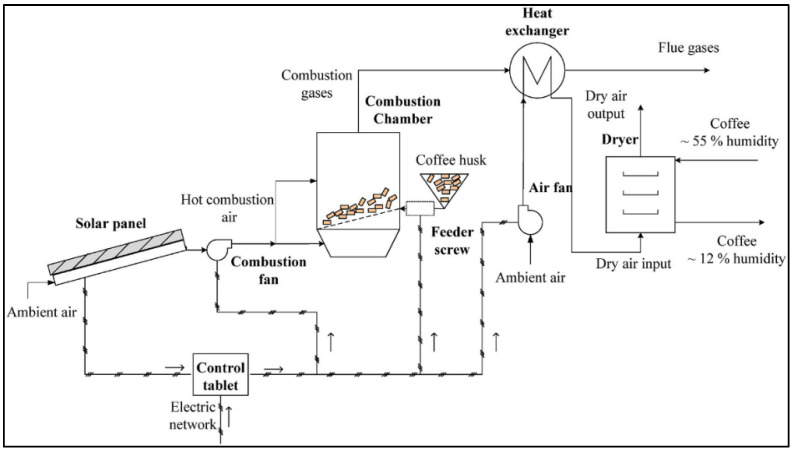
Operating flow diagram of a hybrid drying system. Integration of a biomass combustion chamber (fed by coffee husks) with a forced convection drying unit fed by solar panels for drying parchment coffee [64].

Solar–biomass hybrids are particularly attractive in coffee-producing regions where biomass residues such as husk, parchment, or wood are abundant [63,89]. In these systems, solar radiation provides baseline heating, while a biomass furnace delivers supplementary hot air during periods of low irradiance or high ambient humidity (Figure 15). This combination ensures drying continuity under variable climatic conditions while significantly reducing dependence on fossil fuels [63,189].

Experimental evaluations show that solar–biomass dryers can cut drying times by 40–60% compared to passive solar systems and use up to 50% less biomass than conventional mechanical dryers by leveraging solar pre-heating [66,178]. Furthermore, using coffee husk as fuel creates a closed-loop energy system, enhancing sustainability by valorizing a processing by-product that would otherwise be discarded [163].

Challenges include the need for well-designed furnaces to ensure complete combustion and avoid smoke contamination, which can impart undesirable phenolic or smoky notes to the beans if flue gases mix with the drying air [175]. Proper insulation and air–gas separation are therefore critical to preserve sensory quality.

#### 5.1.3. Solar–Electric Systems

**Figure 16:**
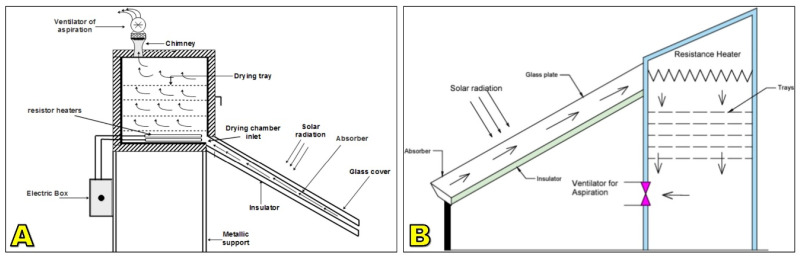
Operating diagram of solar–electric hybrid dryer models. (**A**) Heating of the drying air by manifolds and subsequently, before entering the drying chamber, by electric resistors, under the drying bed [192]. (**B**) Heating of the drying air by manifolds and subsequently, before entering the drying chamber, by electric resistors, above the drying bed [193].

Solar–electric hybrids integrate solar energy with electric resistance heaters or photovoltaic-powered heating elements to stabilize drying conditions [89]. These systems can provide precise temperature control, often within the recommended 40–50 °C range, regardless of fluctuations in solar irradiance [190,191]. Their ability to maintain stable microclimates makes them particularly suitable for specialty coffee production, where consistency and quality preservation are paramount (Figure 16).

Although technically effective, solar–electric dryers are generally more capital- and energy-intensive than other hybrid designs, since they require photovoltaic infrastructure or reliable grid access. Their adoption is therefore limited to demonstration projects, research facilities, or farms targeting high-value specialty markets where premiums justify the higher investment.

To facilitate comparison across hybrid drying technologies, Table 2 summarizes their development stage, economic implications, drying performance, and impact on coffee quality.

### 5.2. Design Principles and Operational Considerations

The effectiveness of hybrid coffee dryers depends not only on the choice of supplemental energy source but also on design principles that govern airflow distribution, heat transfer, and system integration. Proper design ensures that solar energy is maximally harnessed while supplemental inputs are efficiently used to maintain drying continuity and product quality. A central consideration is airflow management. Uniform distribution of heated air through the coffee mass is critical to avoid moisture gradients, case-hardening, or microbial hotspots [170,173]. Hybrid dryers typically use a combination of natural convection (driven by the greenhouse effect) and forced ventilation (from fans or blowers). The placement and size of air inlets, outlets, and vents strongly affect circulation patterns. Computational fluid dynamics (CFD) studies have shown that poorly designed airflow channels can create dead zones where drying stagnates, even if average chamber temperatures are optimal [63,194,195].

Another key principle is energy integration. Supplemental heating, whether from biomass furnaces, electric resistance heaters, or photovoltaic systems, should be designed to complement rather than replace solar input [61,191]. Pre-heating incoming air before it enters the drying chamber is a common strategy, as it raises the temperature and lowers relative humidity, thereby increasing the driving force for evaporation. Insulation of ducts and chambers is equally important to reduce heat losses and improve overall thermal efficiency [136].

Hybrid systems with integrated thermostats or automated controls are better able to regulate temperature fluctuations caused by variable solar irradiance, preventing overheating during midday and under-heating during cloudy conditions [196,197]. Moisture monitoring represents another operational consideration. The heterogeneous nature of coffee batches makes real-time monitoring critical to ensure that beans reach safe moisture content (10–12% wb) uniformly [198]. Hybrid systems are increasingly incorporating digital hygrometers, infrared sensors, or IoT-based monitoring tools, which provide feedback for adjusting airflow or supplemental heating [199,200].

From a practical stance, hybrid dryers must also balance construction costs and maintenance requirements. Systems built with polycarbonate covers and metal frames have greater durability but higher upfront costs, while polyethylene structures are cheaper but degrade quickly under UV exposure. The design must also consider ease of operation and compatibility with locally available materials, especially in smallholder contexts [62,99].

### 5.3. Scalability and Cost Analysis

The scalability of hybrid drying systems depends on the balance between technical performance, capital investment, and operational feasibility. While hybrids consistently outperform traditional and passive solar dryers in terms of speed, uniformity, and safety, their adoption is constrained by economic and infrastructural considerations [62,89,201].

At the smallholder level, hybrid systems are often regarded as too capital-intensive for individual farmers. Construction costs for tunnel- or greenhouse-type dryers with integrated biomass or forced-air components can be 2–5 times higher than those of simple raised beds or patios [63,103,202]. Operational costs also rise when electricity or frequent biomass feeding is required. However, shared cooperative models or community-based infrastructure projects have shown potential to spread investment across multiple producers, increasing access and lowering per-farmer cost [203]. In these contexts, hybrids are attractive because they reduce postharvest losses during adverse weather and help farmers meet international quality and safety standards.

In medium- to large-scale operations, hybrid dryers offer significant energy savings compared to fully mechanical dryers. By relying on solar input during peak hours and supplementing only when irradiance is insufficient, hybrids can reduce fuel consumption by 30–50%, directly lowering operating costs and carbon footprint [64,178]. Biomass-fueled hybrids are particularly well-suited for centralized wet mills that generate large quantities of husk or parchment residues, enabling a closed-loop energy system [204,205,206].

Economic evaluations show that the payback period of hybrid dryers varies widely depending on local energy prices, climate conditions, and market premiums for quality [165,178]. In regions with abundant sunshine, high energy costs, and strong specialty markets, payback periods can be as short as 2–3 years. In less favorable contexts, they may extend beyond 5 years, which limits farmer adoption without external subsidies or development aid [62,63,191].

Scalability also depends on technical capacity and training. Hybrid dryers require more precise operation than traditional systems: airflow must be adjusted, furnaces must be maintained, and covers must be replaced periodically. Without proper management, these systems risk underperforming or even compromising product quality [194,207]. Therefore, long-term adoption is closely tied to extension services, training programs, and institutional support.

In conclusion, hybrid dryers represent a promising pathway toward scalable, sustainable drying solutions that bridge the gap between traditional solar methods and fully mechanical systems. Their long-term viability will depend on design innovations that lower costs, simplify operation, and maximize the use of locally available materials and energy sources. From a practical point of view, no single drying technology is universally optimal. Traditional sun drying is low-cost and widely accessible but highly dependent on weather conditions and prone to quality variability. Mechanical drying provides better process control and more consistent product quality, although it requires higher energy input and investment. Hybrid and solar-assisted systems offer a balance between efficiency and sustainability, but their adoption may be limited by their complexity and cost. Therefore, technology selection depends on the trade-off between quality, energy availability, environmental conditions and economic constraints.

## 6. Emerging and Innovative Drying Technologies

The continuous search for more efficient, sustainable, and quality-preserving drying methods has stimulated research into emerging and innovative technologies that go beyond conventional solar and mechanical systems. These approaches aim to overcome persistent challenges such as long drying times, uneven moisture distribution, high energy demand, and loss of sensory quality. By integrating principles from food engineering, material science, and digital technologies, these methods represent the frontier of coffee drying research [132,208].

Among the most promising innovations are microwave- and infrared-assisted drying, which accelerate moisture removal by directly interacting with water molecules or surface layers of the bean, thereby reducing processing time and energy consumption [209,210]. Other advanced approaches include freeze-drying, primarily used for instant coffee production but increasingly investigated for high-quality preservation, and desiccant-assisted drying, which leverages hygroscopic materials such as silica gel or zeolites to maintain low-humidity environments and enhance drying kinetics [33,42].

In parallel, computational fluid dynamics (CFD) and artificial intelligence (AI) tools are being applied to model, monitor, and optimize drying processes in real time [127,153,211,212]. These approaches provide unprecedented capacity to predict moisture movement, energy balance, and quality outcomes, enabling precision drying strategies that were not feasible with traditional systems. Finally, the integration of innovative dryers with renewable energy sources such as photovoltaic panels and biomass gasifiers is opening new opportunities for sustainable scaling in coffee-producing regions [64].

### 6.1. Microwave Drying

Microwave drying excites water molecules through dipole rotation and ionic conduction (300 MHz–300 GHz), generating volumetric heating that shortens drying times by up to 80% compared to hot air [213,214]. For coffee, this accelerates moisture removal from deep within beans, reducing microbial growth and mycotoxin risks in tropical climates. Hybrid microwave–hot air systems are especially promising, as they combine rapid internal heating with surface stabilization to prevent condensation and mold [215,216].

**Figure 17:**
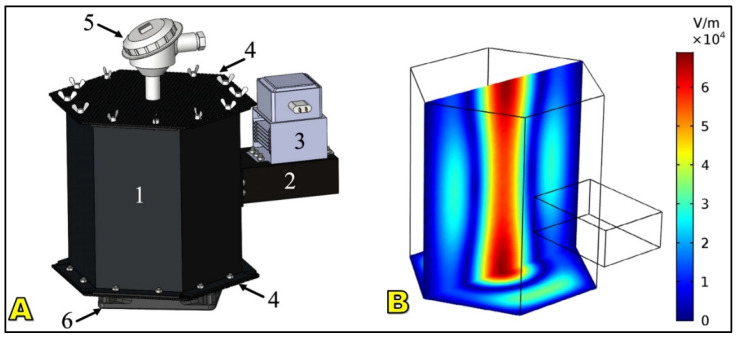
Prototype of microwave coffee dryer. (**A**) 1: hexagonal drying cavity; 2: length waveguide WR340; 3: magnetron 2M319J; 4: perforated cover; 5: calibrated K-type thermocouple; 6: drying air inlet fed by a fan. (**B**) Electric field inside cavity in Simulation [210].

Microwave drying exhibits higher energy efficiency than conventional hot air drying because minimal heat is lost to the surrounding environment, and it reduces external case hardening, resulting in more uniform moisture profiles within the coffee beans [217,218]. However, technical challenges remain, including the risk of localized overheating, microcrack formation, and partial roasting at high power densities, as well as limited penetration depth in dense or high-moisture batches, which complicates uniform large-scale processing [153]. Recent studies emphasize advanced process control approaches, such as pulsed microwave application, monitoring of dielectric properties, and integration of smart sensors (Figure 17), to mitigate hot spots and improve drying uniformity, although high equipment costs and scalability constraints currently restrict industrial adoption despite promising developments in continuous-flow microwave systems [219,220].

### 6.2. Infrared Drying

**Figure 18:**
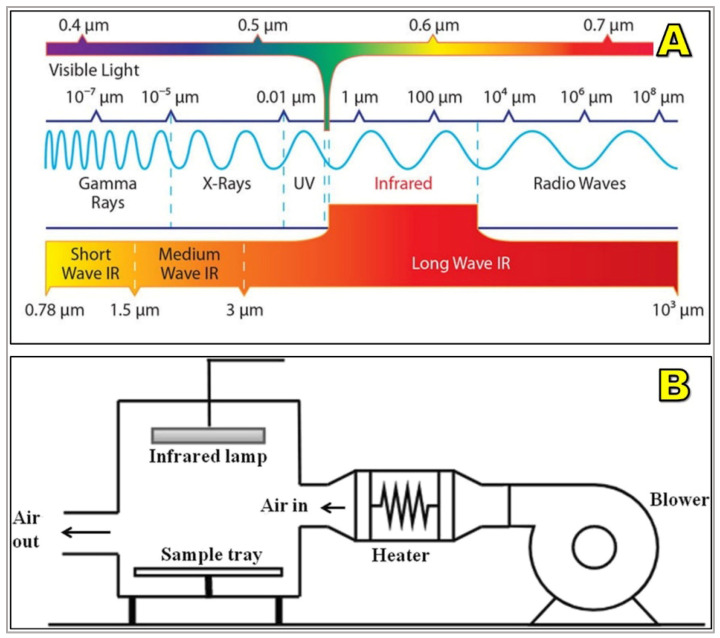
Principles of infrared (IR) drying. (**A**) Electromagnetic spectrum highlighting the Long-Wave IR region utilized for thermal radiation; (**B**) schematic of an experimental hybrid IR-convective dryer featuring an IR lamp, heating element, and centrifugal blower [224].

Infrared (IR) drying transfers energy via radiation (0.78–1000 μm), rapidly heating bean surfaces and expediting evaporation [69,221] (Figure 18). Near-IR penetrates slightly deeper than mid- or far-IR, influencing effectiveness in coffee drying. Compared to convective systems, IR can reduce drying time by 30–60% and achieve thermal efficiencies >50% [222,223].

Its main strength lies in shortening the constant-rate phase, when beans are most susceptible to microbial contamination. As such, IR pre-drying combined with solar or mechanical methods lowers overall drying duration and improves safety under humid conditions [196,225]. However, superficial heating can create sharp moisture gradients, causing surface browning or internal stress cracks [222].

Emerging IR–hot air hybrid systems balance surface and internal drying, preserving organic acids and volatile compounds better than high-temperature convective drying alone [226,227]. Pilot studies in Brazil and Ethiopia have shown that IR drying retains brighter acidity and floral notes compared to hot-air-dried controls, suggesting potential for specialty coffee markets.

### 6.3. Freeze-Drying

**Figure 19:**
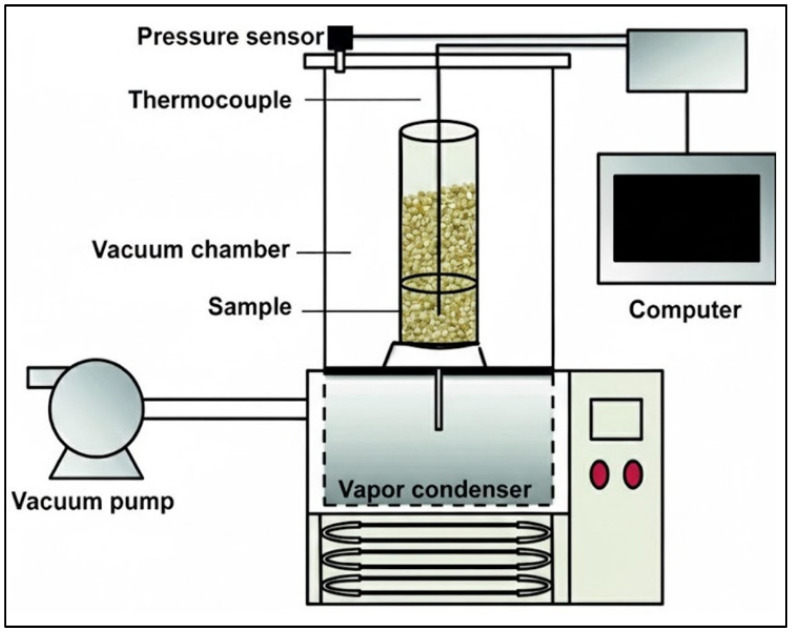
Schematic of an experimental vacuum drying system: Integration of a vacuum chamber, vapor condenser, and real-time monitoring (thermocouple/pressure sensors) to optimize moisture removal at low temperatures, preserving bean chemical precursors [232].

Freeze-drying (lyophilization) removes water by sublimation under vacuum (Figure 19) at sub-zero temperatures (−40 to −50 °C), avoiding liquid-phase transitions that degrade sensitive compounds [228,229]. This method excels in preserving volatile aromatic precursors, sugars, and chlorogenic acids, making it indispensable in instant coffee production [42,230,231].

In coffee, freeze-dried granules dissolve rapidly and retain flavors closer to fresh-brewed coffee compared to spray-dried products [233]. For green beans, experimental trials have shown superior retention of aroma and minimal structural damage, suggesting niche potential for preserving ultra-specialty microlots.

Yet, the process is slow, energy-intensive, and requires costly vacuum/freezing infrastructure, making farm-level adoption unrealistic [234,235]. Current research is exploring energy recovery systems, vacuum-assisted freezing, and microwave freeze-drying hybrids to reduce costs and times, but industrial applications remain confined to soluble coffee production and experimental specialty markets [231].

### 6.4. Desiccant-Assisted Drying

This approach lowers drying air humidity using hygroscopic materials (silica gel, zeolite, activated alumina), enhancing the driving force for moisture diffusion without raising temperature [236,237]. Reported drying time reductions reach 20–40%, while preserving delicate flavor precursors such as organic acids and aldehydes [33,238].

**Figure 20:**
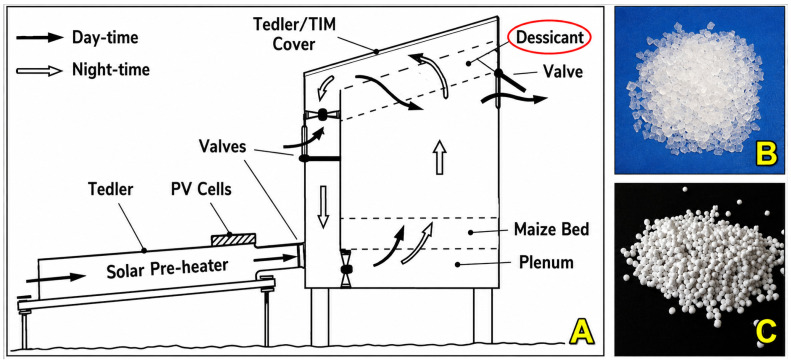
Integrated desiccant-solar drying system: (**A**) Schematic of the solar pre-heater and drying chamber with day/night airflow valves; (**B**,**C**) Solid desiccant materials used for air dehumidification to maintain drying kinetics during high-humidity periods [242].

It is particularly valuable under humid tropical conditions, where solar drying stagnates. Desiccant-assisted systems maintain drying continuity even during rainfall or nighttime, when microbial risks are highest. Hybrid solar–desiccant dryers tested in Southeast Asia have allowed safe 24 h drying cycles, improving efficiency and reducing spoilage losses [239,240] (Figure 20).

Challenges include desiccant regeneration, which requires periodic heating or solar exposure, adding complexity and costs. Advanced zeolite-based systems offer higher adsorption but remain too expensive for smallholders. Research is ongoing into low-cost natural desiccants such as clay minerals or agricultural residues treated for porosity, which could democratize the technology [237,241].

### 6.5. Computational and Intelligent Systems

The digitalization of drying science has catalyzed a paradigm shift in coffee post-harvest processing, transitioning from empirical observation to high-fidelity Computational Fluid Dynamics (CFD) and Multiphysics Modeling [243]. By simulating complex airflow patterns and hygrothermal gradients within tunnel, solar, and hybrid mechanical dryers, CFD facilitates the precise identification of stagnant zones and the structural optimization of dryer geometries [10,63,127]. These computational frameworks are instrumental in enhancing drying uniformity and thermodynamic efficiency, effectively mitigating energy dissipation while preserving the biochemical integrity of the bean, a critical factor for both product quality and process sustainability in specific microclimatic contexts [153,244].

Simultaneously, Artificial Intelligence (AI) and Machine Learning (ML) architectures, including neural networks and deep learning, have superseded classical models in predicting the non-linear drying kinetics of biological matrices [211]. Unlike deterministic equations, these stochastic algorithms process experimental data to forecast equilibrium moisture content and terminal sensory profiles with high granularity [212,245]. When integrated with Internet of Things (IoT) platforms and non-destructive smart sensors (e.g., infrared and dielectric), these systems enable real-time autonomous control [200,246]. Such adaptive infrastructures facilitate precision drying by adjusting operational parameters in situ, thereby preventing physiological defects like case hardening or microbial proliferation through remote, cloud-based monitoring [199,201].

Despite these techno-scientific advancements, the deployment of intelligent drying systems within smallholder-dominated sectors is hindered by high capital expenditure, infrastructural deficits, and technical barriers [247,248]. The democratization of precision drying necessitates a transition toward cooperative-led digital platforms and participatory technology transfer models that pool computational resources [249,250]. Ultimately, the long-term viability of these digitalized ecosystems depends upon their convergence with renewable energy frameworks. This holistic integration ensures that the evolution of data-driven decision-making remains commensurate with the decarbonization and economic resilience of the global coffee value chain [72,182,251].

Emerging drying technologies offer significant potential to improve drying efficiency, precision, and quality preservation, but most remain at pilot, laboratory, or niche-commercial stages. Their current relevance lies less in immediate widespread adoption than in the technological directions they reveal: lower-temperature control, smarter process monitoring, improved moisture uniformity, and tighter integration of energy management with product-quality preservation. Their future value will depend on whether these advances can be translated into systems that are technically robust, economically accessible, and adaptable to coffee-producing regions with limited infrastructure.

To facilitate comparison among emerging drying technologies, Table 3 summarizes their development stage, applicability, economic considerations, drying performance, and impact on coffee quality.

## 7. Sustainability, Scalability, and Future Perspectives

Sustainability and scalability have become central considerations in the evaluation of coffee drying technologies, as drying represents one of the most energy-intensive and environmentally impactful stages of postharvest processing. Beyond achieving target moisture content and preserving sensory quality, drying systems must balance energy efficiency, carbon emissions, operational costs, and resource availability. These factors are increasingly relevant under conditions of climate variability, rising energy prices, and growing pressure from markets and certification schemes to demonstrate environmentally responsible production practices.

At the same time, the future of coffee drying depends on the ability of technologies to scale across highly heterogeneous production contexts, ranging from smallholder farms to industrial processing facilities. Differences in infrastructure, access to capital, labor availability, and regional climate strongly influence technology adoption and performance. Addressing these challenges requires not only technical innovation but also socio-economic and institutional approaches that support equitable access, adaptive system design, and long-term resilience. Climatic conditions influence coffee bean development, affecting attributes such as density, porosity, and chemical composition; however, their relationship with bean size is indirect and highly dependent on genotype and agronomic management. From a drying perspective, these structural and compositional factors play a more critical role than size alone in determining heat and mass transfer behavior. Together, these dimensions define the pathway toward drying solutions that are both technically robust and aligned with sustainability goals across the global coffee sector.

### 7.1. Energy Use and Environmental Impacts

Drying is one of the most energy-intensive stages in the postharvest chain of coffee, and the choice of technology directly influences both production costs and environmental sustainability [72,252]. Mechanical dryers typically require between 3 and 6 MJ per kilogram of water removed, with static bed and cross-flow designs consuming more energy due to prolonged cycles and inefficient airflow. Rotary and drum dryers, while faster, demand continuous fuel combustion, often relying on fossil fuels or unsustainably harvested firewood [60,149]. By contrast, solar and hybrid systems significantly reduce external energy dependence, though their performance is constrained by climatic variability [182,251].

Several strategies have been proposed to improve energy efficiency, including airflow optimization, chamber insulation, exhaust heat recovery, and integration of renewable sources such as photovoltaic-assisted fans or biomass gasifiers [64,136]. However, each approach must be evaluated within a life cycle assessment (LCA) framework to capture trade-offs between operational savings, embodied energy, and material use. Studies indicate that hybrid solar–biomass systems can cut emissions by 30–50%, while PV-assisted systems can approach zero operational emissions, albeit with high embodied energy in panels and batteries [63,253,254]. Ultimately, long-term sustainability requires balancing energy efficiency with carbon reduction and resource availability at the local level.

### 7.2. Adoption in Smallholder Contexts and Regional Experiences

Most of the global coffee production is carried out by smallholder farmers cultivating less than five hectares, who often face barriers to adopting advanced drying systems [248,255]. Mechanical dryers, despite their efficiency, demand significant capital investment, reliable electricity, and technical maintenance—all of which are limited in rural contexts. Cooperative-level initiatives and NGO support have proven critical for bridging these gaps by enabling cost-sharing, training, and collective access to equipment [250].

Case studies illustrate regional differences in adoption dynamics. In Latin America, parabolic houses and polycarbonate-covered solar dryers supported by cooperatives have improved quality consistency and shortened drying times [49,62,94]. In Brazil, rotary and silo dryers dominate large-scale production, reflecting both industrial capacity and market incentives. In Africa, raised beds remain the norm, but hybrid solar–biomass dryers show promise in reducing ochratoxin A risk in humid highlands [47,87]. In Asia, particularly Vietnam and Indonesia, hybrid systems and biomass gasifiers using coffee husk and parchment highlight the potential for context-adapted solutions [61,132,256].

Across regions, three lessons emerge: (1) collective investment models are crucial for scaling, (2) specialty coffee markets provide stronger incentives for innovation, and (3) climatic context dictates the suitability of each technology. These experiences demonstrate that sustainability and scalability require locally adapted solutions rather than universal prescriptions.

### 7.3. Technical and Scientific Challenges

Despite technological progress, the drying of coffee remains poorly understood at multiple scales. Most models oversimplify bean heterogeneity, anisotropy, and the coupling of heat and mass transfer. Multiscale modeling frameworks that integrate intracellular water dynamics with bulk bed behavior are needed to guide dryer design and process optimization [135,257,258]. Likewise, low-cost real-time monitoring tools for moisture, water activity, and airflow distribution remain scarce, limiting precise process control [200,259,260]. Emerging IoT-based systems and sensor networks could offer affordable solutions but require further adaptation for smallholder use.

To provide a clearer perspective on the trade-offs between conventional and advanced drying technologies, Table 4 presents a comparative analysis of their energy consumption, environmental impact, and operational performance.

### 7.4. Emerging Innovations and Research Gaps

Novel technologies such as microwave, infrared, freeze-drying, and desiccant-assisted systems have demonstrated potential for reducing drying times and preserving flavor precursors, but their application in coffee remains at pilot or lab scale. Economic feasibility, scalability, and social acceptance are still uncertain. Integration of Artificial Intelligence (AI) and Computational Fluid Dynamics (CFD) for predictive control represents another frontier, with early studies showing promise in optimizing airflow and heat distribution.

From a sustainability perspective, life cycle assessments of dryer technologies are still limited. Few studies evaluate embodied energy, durability, and recyclability of materials, even though these factors determine the long-term viability of “green” dryers. Research should prioritize holistic evaluations that consider not only technical efficiency but also environmental and social trade-offs.

### 7.5. Future Outlook

The future of coffee drying will depend on the ability to combine technical efficiency with economic accessibility and environmental responsibility. Priority should be given to the development of low-cost sensor systems capable of supporting real-time control of temperature, airflow, and moisture content under field conditions. At the same time, multiscale modeling approaches should be expanded to better connect bean-level heat and mass transfer phenomena with full-system dryer performance. Greater integration of renewable energy sources, particularly solar energy and biomass residues generated within coffee processing systems, will be essential for reducing dependence on fossil fuels and improving resilience under volatile energy markets. Equally important is the strengthening of cooperative models, extension services, and policy instruments that enable smallholders to access improved drying technologies. Progress in coffee drying will therefore depend not only on engineering advances but also on institutional and socio-economic frameworks capable of translating innovation into broad and durable adoption.

## 8. Conclusions

Coffee drying remains a critical determinant of post-harvest stability and sensory excellence, representing a complex intersection of thermodynamic efficiency and biochemical preservation. While traditional patio and raised-bed systems persist due to their low capital intensity, their inherent vulnerability to climatic stochasticity and microbial risks necessitates a transition toward controlled drying environments. The evolution from passive solar structures to active mechanical and hybrid systems, integrating infrared, microwave, and desiccant-assisted technologies, demonstrates a clear trajectory toward balancing accelerated drying kinetics with the stringent requirements of specialty markets and international safety standards.

The efficacy of these drying systems is intrinsically linked to the physical and chemical transformations within the bean, which are governed by a multi-scale interaction between environmental variables, varietal characteristics, and processing methodologies. Sustainability assessments reveal that the adoption of advanced drying technologies in smallholder contexts is not merely a technical challenge but a socio-economic one, where the trade-offs between operational performance and accessibility are decisive. Future research must, therefore, prioritize the development of low-cost sensor networks and refined heat and mass transfer models that can be seamlessly integrated into cooperative-level infrastructures, ensuring consistency across diverse production scales.

Looking forward, the resilience of the coffee value chain depends on the systematic integration of renewable energy sources and the refinement of on-field management practices to mitigate the impacts of climate change and rising energy costs.

Overall, the effectiveness of coffee drying systems lies in their ability to balance drying efficiency, product quality and resource use. Rather than a single optimal solution, the most suitable technology depends on local conditions, scale of production and desired quality attributes. Future research should focus not only on the integration of process control, real-time monitoring, and energy-efficient designs to optimize drying performance while ensuring sustainability and adaptability to varying climatic conditions, but also on democratizing access to precision drying through participatory technology transfer and socio-economic frameworks that align technical performance with the realities of rural producers. Ultimately, fostering cross-disciplinary collaboration will be essential to developing adaptive, low-carbon drying systems that secure the economic viability and environmental sustainability of global coffee production in an increasingly volatile market.

## Figures and Tables

**Table 1:** Comparison of coffee drying systems with respect to drying time, energy efficiency, environmental exposure, and impacts on physical and sensory quality.

Drying System	Typical Drying Time	Climate Dependence	Investment/ Operating Cost	Energy Efficiency	Contamination Risk	Impact on Quality Outcomes
Patios	7–20 days	Very high; directly affected by rain, RH, and solar radiation	Very low; simple cement or asphalt floor	Low; relies on direct solar and conduction from surface	High; exposure to soil, dust, and rainwater	Variable; can yield acceptable profiles in dry climates but often heterogeneous moisture and defects
Raised (African) beds	8–18 days	High; still weather-dependent, though airflow improves resilience	Low–moderate; requires wood/metal frames and mesh	Moderate; bidirectional airflow enhances convective drying	Moderate; reduced ground contact but vulnerable to rain unless sheltered	Generally higher cup scores than patios; cleaner profiles, reduced off-notes
Passive solar dryers	7–12 days	Moderate; greenhouse effect buffers climate variability	Moderate; requires plastic or polycarbonate covers	Moderate–high; internal temps 5–20 °C above ambient	Low; protection from dust, soil, and rain	Improved uniformity, reduced defects, higher stability in storage
Active solar dryers	4–7 days	Low; forced ventilation ensures consistent airflow	High; requires collectors, fans, or PV panels	High; efficient heat/mass transfer, reduced drying time	Very low; controlled microclimate minimizes microbial growth	Superior uniformity, higher cupping scores, cleaner and more consistent profiles

**Table 2:** Comparative overview of hybrid coffee drying systems.

Hybrid System Type	Development Stage	Capital Cost	Operating Cost	Drying Rate	Quality Performance	Key Advantages	Main Limitations
Solar–Mechanical	Pilot to commercial (small–medium scale)	Moderate	Low–moderate (electricity for fans)	↑ 30–40% vs. solar	Improved uniformity; reduced microbial risk	Better airflow control; relatively simple integration	Dependence on electricity; added equipment cost
Solar–Biomass	Pilot to commercial (small–large scale)	Moderate	Low (uses residues as fuel)	↑ 40–60% vs. solar	Good quality if combustion is well controlled	Utilizes local biomass; reduced fossil fuel use; closed-loop systems	Risk of smoke contamination; requires furnace design and management
Solar–Electric	Laboratory to pilot (limited commercial use)	High	Moderate–high (electricity demand)	High and stable	Excellent control; suitable for specialty coffee	Precise temperature control; independence from climate variability	High capital cost; requires infrastructure (PV or grid); limited adoption

**Table 3:** Comparative overview of emerging and innovative coffee drying technologies.

Technology	Development Stage	Applicability	Capital cost	Drying Performance	Quality-Related Outcomes	Key Advantages	Main Limitations
Microwave drying	Laboratory to pilot (limited industrial trials)	Experimental, specialty processing	High	Very fast; high energy efficiency	More uniform internal moisture; reduced case hardening	Rapid volumetric heating; high efficiency	Risk of overheating; microcracks; limited penetration depth; scalability challenges
Infrared (IR) drying	Pilot to early commercial	Pre-drying or hybrid systems	Moderate	30–60% reduction in drying time; efficiency >50%	Better retention of acidity and volatile compounds	Fast surface heating; improved safety under humid conditions	Surface overheating; moisture gradients; risk of internal stress
Freeze-drying	Commercial (instant coffee); experimental for green beans	Industrial (soluble coffee); niche specialty applications	Very high	Very slow; highly energy-intensive	Excellent preservation of aroma, sugars, and acids	Superior quality retention; minimal structural damage	Very high cost; complex infrastructure; not suitable for farm-level use
Desiccant-assisted drying	Pilot to early commercial	Humid/tropical regions; hybrid systems	Moderate	20–40% reduction in drying time	Preserves delicate flavor precursors (organic acids, aldehydes)	Enables drying under high humidity; continuous operation	Requires desiccant regeneration; added system complexity
Computational and intelligent systems (AI/CFD/IoT)	Laboratory to pilot (emerging adoption)	Cross-cutting (all dryer types)	High (initial investment)	Optimized drying kinetics; improved efficiency	Enhanced uniformity; reduced defects; better quality control	Real-time monitoring; predictive modeling; precision drying	High cost; technical complexity; limited accessibility for smallholders

**Table 4:** Comparative analysis of traditional and advanced coffee drying methods in terms of energy use, sustainability, and performance.

Drying Method	Energy Consumption	Carbon Footprint	Sustainability	Drying Performance	Scalability	Key Remarks
Traditional (open sun/patios/raised beds)	Very low (solar-dependent)	Very low	High (minimal external inputs)	Slow; highly climate-dependent; non-uniform	High (smallholder level)	Low cost but high risk of contamination and quality variability
Solar dryers (improved passive systems)	Low	Very low	High	Moderate; improved uniformity vs. open sun	High (small–medium scale)	Limited by weather variability; relatively low investment
Mechanical dryers (static, cross-flow, rotary, drum)	High (≈3–6 MJ/kg water removed)	High (fossil fuel dependent)	Low–moderate	Fast; high control and reliability	High (medium–industrial scale)	High cost and emissions; consistent performance
Hybrid systems (solar–mechanical, solar–biomass, solar–electric)	Moderate	Moderate–low (30–50% emission reduction possible)	Moderate–high	Faster than solar; more stable performance	Medium–high	Balanced solution; reduced fuel dependency; higher initial cost
Emerging technologies (microwave, IR, freeze-drying, desiccant, AI-integrated)	Variable (often high or optimized)	Variable (technology-dependent)	Potentially high (future-oriented)	Very fast or highly controlled; high precision	Low (currently limited to pilot/industrial niche)	High cost; scalability and accessibility remain key challenges

## Data Availability

No new data were created or analyzed in this study. Data sharing is not applicable to this article.

## Abbreviations

AI	Artificial Intelligence
CFD	Computational Fluid Dynamics
IR	Infrared
IoT	Internet of Things
LCA	Life Cycle Assessment
ML	Machine Learning
PV	Photovoltaic
RH	Relative Humidity
wb	Wet Basis
